# The effectiveness of different exercise modalities on sleep quality: A protocol for systematic review and network meta-analysis

**DOI:** 10.1097/MD.0000000000021169

**Published:** 2020-07-17

**Authors:** Peiye Cao, Ying Cai, Shifang Zhang, Qiaoqin Wan

**Affiliations:** a Peking University People's Hospital; b School of Nursing, Peking University, Beijing, China.

**Keywords:** effectiveness, exercise, network meta-analysis, sleep quality, systematic review

## Abstract

**Background:**

Recently, the effect of exercise on sleep quality has been paid increasing attention by researchers. A number of systematic reviews and direct meta-analyses have explored the effectiveness of exercise on sleep quality. However, which type of exercise is optimal for sleep quality is still not clear. Therefore, this study aims to adopt network meta-analysis to evaluate the effect of different exercise modalities on sleep quality and find the most effective one for improving sleep quality.

**Methods:**

The databases searched included PubMed, Web of Science, EMBASE, CENTRAL, PsycINFO, and SPORTDiscus. Studies published in English and from the dates of database inception to October 8, 2019, were included. Besides, we also checked the reference lists of systematic reviews published in recent years. Two independent researchers screened all studies, included the studies that met eligible criteria and will then extract data. The risk of bias will be assessed by the 2 researchers according to the modified Cochrane Risk of Bias Tool. During these processes, any disagreement will be discussed to reach a consensus. STATA 14.0 software will be used for direct meta-analysis and network meta-analysis.

**Results:**

High-quality synthesis of current evidences will be provided from Pittsburgh sleep quality index. And the effectiveness of different exercise modalities for sleep quality will also be shown.

**Conclusion:**

This study will provide the evidence of the most effective exercise for better sleep quality.

**Registration:**

This study was registered with the International Platform of Registered Systematic Review and Meta-Analysis Protocols (registration number: INPLASY202050096).

## Abstract 2

Supplemental Digital Content is available in the text

## 1 Introduction

Sleep occupies a substantial proportion of life and is closely related to mental or physical health. However, not everyone has good sleep quality. There are 32.1% people in Netherlands with sleep disturbance.^[1]^ And the prevalence of sleep disorder increases with age; 3.2% among children under 10 years, 5.2% among young adults, and 22.3% among seniors aged 75 or order.^[2]^ As for the effect of sleep on people, people with good sleep quality tend to have good health and wellbeing while those with sleep disturbance may suffer from the increased risk of cardiovascular disease,^[3,4]^ dementia, depression, and immune disorders.^[5]^ Additionally, poor sleep quality can result in day-time sleepiness,^[6]^ which leads to misjudgment, inattention and low work efficiency. Accordingly, how to improve sleep quality has been a great concern.

There are many ways to enhance sleep quality. Among them, exercise as one of the non-pharmacological methods has been paid increasing attention by researchers. Regular exercise was listed as a recommendation for better sleep.^[7]^ Also, plenty of studies revealed that exercise improved sleep quality.^[8,9]^ During the exercise, melatonin secretion as well as the release of neurotrophic factors increase, Aβ concentration decreases and inflammation level is regulated, which will probably affect sleep quality.^[8,10]^ What is more, a number of systematic reviews and direct meta-analysis have explored the effectiveness of exercise on sleep quality.^[11–14]^ However, which type of exercise is optimal for sleep quality cannot be concluded from direct meta-analysis. Superior to direct meta-analysis, network meta-analysis can compare and analyze multiple exercise modalities’ effects on sleep quality simultaneously. A recent network meta-analysis was published to evaluate the effect of different meditation exercises on sleep quality in older people.^[15]^ In this review, it only focused on older people and only involved meditation exercises without consideration of any other kinds of exercise as well as specific dimensions of sleep quality. Therefore, a network meta-analysis needs to be conducted to systematically assess the effect of different exercise modalities (e.g., aerobic exercise, resistance exercise and mind-body exercise) on sleep quality and identify the most effective exercise for improving sleep quality.

## 2 Methods

This protocol was completed following the guidance in Preferred Reporting Items for Systematic Reviews and Meta-Analysis Protocols.^[16]^ And it was registered with the International Platform of Registered Systematic Review and Meta-Analysis Protocols (registration number INPLASY202050096).

### 2.1 Selection criteria

#### 2.1.1 Study design

Randomized controlled trials in English were included. Studies without full-text articles were excluded.

#### 2.1.2 Population

All people with or without sleep problems.

#### 2.1.3 Intervention

The studies involved multiple exercise modalities including aerobic exercise, anaerobic exercise, resistance exercise, combined exercise, mind-body exercise, and flexibility exercise. Aerobic exercise refers to any exercise that body's large muscle moves in a rhythmic manner for a while,^[17]^ which improves cardiopulmonary endurance (e.g., walking, running). Anaerobic exercise is a high intensity and instantaneous exercise (VO_2_max is low).^[18]^ Resistance exercise is defined as an exercise making muscles work or hold against an externally imposed force that can increase muscle strength, endurance, or power (e.g., elastic band training).^[19]^ Combined exercise means that participants perform 2 or more types of exercise at the same time. Mind-body exercise is a combination of physical activity and mediation (e.g., yoga, Pilate, Tachi). Flexibility exercise is characterized as stretching and relaxation. There was no limitation on the exercise intensity, duration, and setting. Besides, the intervention implementation and supervision were not restricted as well.

#### 2.1.4 Control

Control group included any other forms other than the same exercise as the intervention group, such as other forms of exercise, health education, usual care, and social activity.

#### 2.1.5 Outcomes

Sleep quality was the only outcome. We chose Pittsburgh sleep quality index (PSQI) to measure sleep quality, considering that it was an internationally recognized tool for assessing sleep quality and was widely used in sleep medicine research. PSQI comprises 7 components: subjective sleep quality, sleep duration, sleep latency, sleep disturbances, habitual sleep efficiency, use of sleep medication, and daytime dysfunction. We focused on the change in both total score of PSQI and score of each component between experimental group and control group from baseline to the end of intervention.

### 2.2 Data sources and search strategy

We developed search strategy based on population, intervention, comparison, outcome, and study design (PICOS). After the review by experts, we searched the electronic database, including PubMed, web of science, EMBASE, Cochrane Central Register of Controlled Trials, SPORTDiscus, and PsycINFO and studies from database inception to October 8, 2019, were included. The specific search strategies for each database are shown in Supplementary File 1. To ensure that all relevant literatures were included, we also checked the reference lists of systematic reviews published in recent years.

### 2.3 Study selection

All studies were imported into EndNote. First, we removed the duplicate studies. Secondly, 2 independent researchers (Cai & Cao) screened titles and abstracts to remove irrelevant studies. Then full texts of remaining studies were screened to determine ultimate studies. During the selection, any disagreement between the 2 independent researchers were resolved by discussion or the third researcher (Zhang).

### 2.4 Data extraction

Data will be extracted by 2 researchers independently (Cai & Cao) and recorded in a Microsoft Excel that includes title, the first author, country, number of participants, participants’ mean age, intervention mode, intervention time, intervention frequency, intervention duration, control mode, and outcome measure. If there is any inconsistency, the 2 researchers will discuss or the third researcher (Zhang) will appear to eliminate the inconsistency. Normally, we will extract mean ± standard deviation of the outcome. When mean and standard deviation cannot be found in the article, we will transform the existing data according to the Cochrane handbook and previous studies^[20–22]^ or contact the corresponding authors. The specific data transformations are shown in Supplementary File 2.

### 2.5 Risk of bias assessment

Two researchers (Cai & Zhang) will assess the risk of bias independently based on the modified Cochrane Risk of Bias Tool,^[23]^ including random sequence generation, allocation concealment, blinding of participants, and researchers, blinding of outcome evaluator, incomplete outcome data addressed, selective reporting of results, and other risk bias. Finally, all eligible studies will be identified as “high risk of bias”, “unclear risk of bias” and “low risk of bias” according to the results of each item evaluation. If there is any disagreement, the 2 researchers will discuss it or the third researcher (Cao) will appear to resolve it. Finally, Review Manager 5.3 software will be used to present the risk of bias graph.

### 2.6 Statistics analysis

Literature data such as publication year, authors, and sample size of groups will be input into STATA 14.0 software for network and direct meta-analysis. A *P*-value (2-tailed) less than .05 will be considered statistically significant. Standard mean differences with 95% confidence intervals will be calculated as the scores of PSQI is continuous data.

For the direct meta-analysis, the random effect model will be used if there was a significant difference between study heterogeneity; otherwise, the fixed effect model will be used. Statistical heterogeneity will be considered significant if I^*2*^ > 50% and *P* value of Cochran's Q statistic < .10.^[24,25]^

Network meta-analysis will be made using the function of “networkplot” in STATA 14.0 software. It divides into 5 steps.^[26]^ The first step is to draw a network plot to understand which interventions are compared directly, how comparations flow indirectly and the contribution of various interventions.^[27]^ Nodes and lines are connected to present direct comparations in included studies. The size of the nodes and the width of the lines are used to describe the contribution of different forms of interventions. The second step will generate a contribution plot. The contribution of each direct comparison to each network estimate is calculated and summarized in the plot.^[28]^ The third step is to investigate the 2 parts of inconsistency.^[29]^ One is global inconsistency that is assessed by comparison of the fit and parsimony of the inconsistency and consistency models via the Wald test. The *P* value should be higher than .05 if there is no statistically significant global inconsistency.^[30]^ The other is local inconsistency that is assessed by calculation of the differences between direct and indirect estimates in every closed loop within the network.^[31]^ The 95% confidence intervals of inconsistency factor in each closed loop should be truncated at 0 if there is no statistically significant local inconsistency.^[30]^ The fourth step will create the network forest plot to compare the summary size of effectiveness among different interventions. The last step is to determine relative rankings of interventions. The superiorities of different interventions are calculated and reported as the surface under the cumulative ranking curve (SUCRA),^[32]^ ranking from 1 (indicating that the intervention has a high probability of being best) to 0 (indicating that the intervention has a high probability of being worst). A high surface under the cumulative ranking curve rank corresponds with a high ranking of the improvement of sleep quality compared with other exercise modalities.

To evaluate publication bias in the network meta-analysis, a “comparison-adjusted” funnel plot will be drawn.^[33]^ If the funnel plot is symmetrical near the 0 line, it indicates that there is no publication bias.

Subgroup analyses will also be planned to find the discrepancy for specific populations and intervention dimensions (including time, frequency, and duration). The subgroup factors will be as follows:

(1) age (using age 60 years as a cut-off point according to World Health Organization),
(2) intervention time (using 60 minutes as a cut-off point),^[34]^
(3) intervention frequency (using 3 times a week as a cut-off point),^[35]^ and
(4) intervention duration (using 6 months as a cut-off point).^[18]^

## 3 Discussion

Sleep is a basic human need and it is crucial to our overall health as well as day-to-day functioning. Exercise as a healthy lifestyle was recommended to promote sleep quality. Recently, many studies and systematic reviews have explored the effectiveness of exercise on sleep quality, but the different exercise modalities’ effects are still unclear. To compare different exercise modalities’ effects on sleep quality and give the evidence of the most effective exercise, this study will be conducted. This study will be performed according to Preferred Reporting Items for Systematic Reviews and Meta-Analysis Protocols.

### 3.1 Ethics and dissemination

Ethical approval and participant consent are not required. The results will be disseminated by publication in a peer-reviewed journal. This study will provide the evidence of the different exercise modalities’ effectiveness on sleep quality for clinical practice and future research.

## Author contributions

Peiye Cao: Study selection, data extraction, quality assessment, statistical analysis. Ying Cai: Study selection, data extraction, quality assessment, manuscript drafting.

Shifang Zhang: Study selection, data extraction, quality assessment.

Qiaoqin Wan: Study design, manuscript drafting.

All authors reviewed and approved the final version.

**Conceptualization:** Peiye Cao, Qiaoqin Wan.

**Data curation:** Ying Cai, Peiye Cao, Shifang Zhang.

**Formal analysis:** Peiye Cao.

**Methodology:** Ying Cai, Peiye Cao, Shifang Zhang.

**Project administration:** Ying Cai, Peiye Cao.

**Resources:** Ying Cai, Peiye Cao.

**Software:** Peiye Cao.

**Supervision:** Qiaoqin Wan.

**Validation:** Ying Cai, Peiye Cao, Shifang Zhang, Qiaoqin Wan.

**Visualization:** Ying Cai, Peiye Cao, Shifang Zhang.

**Writing – original draft:** Ying Cai, Peiye Cao.

**Writing – review & editing:** Qiaoqin Wan.

## Supplementary Material

Supplemental Digital Content

## Supplementary Material

Supplemental Digital Content

## References

[1] Kerkhof GA Epidemiology of sleep and sleep disorders in the Netherlands. Sleep Med 2017;30:229–39. 28215254 10.1016/j.sleep.2016.09.015

[2] Fund N Green A Chodick G The epidemiology of sleep disorders in Israel: results from a population-wide study. Sleep Med 2020;67:120–7. 31918117 10.1016/j.sleep.2019.10.010

[3] Chair SY Wang Q Cheng HY Relationship between sleep quality and cardiovascular disease risk in Chinese post-menopausal women. BMC Womens Health 2017;17:79–179. 28893224 10.1186/s12905-017-0436-5 PMC5594540

[4] Lo K Woo B Wong M Subjective sleep quality, blood pressure, and hypertension: a meta-analysis. J Clin Hypertens (Greenwich) 2018;20:592–605. 29457339 10.1111/jch.13220 PMC8031314

[5] Gulia KK Kumar VM Sleep disorders in the elderly: a growing challenge. Psychogeriatrics 2018;18:155–65. 29878472 10.1111/psyg.12319

[6] Guilleminault C Partinen M Maria Antonia Q-S Determinants of daytime sleepiness in obstructive sleep apnea. Chest 1988;94:32–7. 3383654 10.1378/chest.94.1.32

[7] Global Council on Brain Health. The Brain-Sleep Connection: GCBH Recommendations on Sleep and Brain Health. 2016. Available at www.GlobalCouncilOnBrainHealth.org.

[8] Al-Sharman A Khalil H El-Salem K The effects of aerobic exercise on sleep quality measures and sleep-related biomarkers in individuals with Multiple Sclerosis: a pilot randomised controlled trial. NeuroRehabilitation 2019;45:107–15. 31403958 10.3233/NRE-192748

[9] Aibar-Almazan A Hita-Contreras F Cruz-Diaz D Effects of Pilates training on sleep quality, anxiety, depression and fatigue in postmenopausal women: a randomized controlled trial. Maturitas 2019;124:62–7. 31097181 10.1016/j.maturitas.2019.03.019

[10] Garner JM Chambers J Barnes AK Changes in brain-derived neurotrophic factor expression influence sleep-wake activity and homeostatic regulation of rapid eye movement sleep. Sleep 2018;41:1–4. 10.1093/sleep/zsx194 PMC6018753 29462410

[11] Kovacevic A Mavros Y Heisz JJ The effect of resistance exercise on sleep: a systematic review of randomized controlled trials. Sleep Med Rev 2018;39:52–68. 28919335 10.1016/j.smrv.2017.07.002

[12] Lederman O Ward PB Firth J Does exercise improve sleep quality in individuals with mental illness? J Psychiatr Res 2019;109:96–106. 30513490 10.1016/j.jpsychires.2018.11.004

[13] Mercier J Savard J Bernard P Exercise interventions to improve sleep in cancer patients: a systematic review and meta-analysis. Sleep Med Rev 2017;36:43–56. 27939642 10.1016/j.smrv.2016.11.001

[14] Vanderlinden J Boen F van Uffelen JGZ Effects of physical activity programs on sleep outcomes in older adults: a systematic review. Int J Behav Nutr Phys Act 2020;17:11–25. 32024532 10.1186/s12966-020-0913-3 PMC7003368

[15] He B Zhang L Zhuang JH The effects of different meditation exercises on sleep quality in older people: a network meta-analysis. Eur Geriatr Med 2019;10:543–52. 10.1007/s41999-019-00212-1 34652740

[16] Moher D Shamseer L Clarke M Preferred Reporting Items for Systematic Review and Meta-Analysis Protocols (PRISMA-P) 2015 statement. Syst Rev 2015;4:1–1. 25554246 10.1186/2046-4053-4-1 PMC4320440

[17] U.S. Department of Health and Human Services. Physical Activity Guidelines for Americans. 2nd ed Washington, DC: U.S. Department of Health and Human Services; 2018.

[18] Pan B Ge L Xun YQ Exercise training modalities in patients with type 2 diabetes mellitus: a systematic review and network meta-analysis. Int J Behav Nutr Phys Act 2018;15:72–85. 30045740 10.1186/s12966-018-0703-3 PMC6060544

[19] Busch AJ Webber SC Richards RS Resistance exercise training for fibromyalgia. Cochrane Database Syst Rev 2013;2013:CD010884–110884. 10.1002/14651858.CD010884 PMC6544808 24362925

[20] Hozo SP Djulbegovic B Hozo I Estimating the mean and variance from the median, range, and the size of a sample. BMC Med Res Methodol 2005;5:13–13. 15840177 10.1186/1471-2288-5-13 PMC1097734

[21] Wan X Wang W Liu J Estimating the sample mean and standard deviation from the sample size, median, range and/or interquartile range. BMC Med Res Methodol 2014;14:135–135. 25524443 10.1186/1471-2288-14-135 PMC4383202

[22] Higgins J Thomas J Chandler J Cochrane Handbook for Systematic Reviews of Interventions version 6.0 (updated July 2019). 2nd ed. Chichester, UK: John Wiley & Sons; 2019.

[23] Higgins JPT Altman DG Gøtzsche PC The Cochrane Collaboration's tool for assessing risk of bias in randomised trials. BMJ 2011;343:d5928–15928. 22008217 10.1136/bmj.d5928 PMC3196245

[24] Higgins JP Thompson SG Quantifying heterogeneity in a meta-analysis. Statistics in medicine 2002;21:1539–58. 12111919 10.1002/sim.1186

[25] Higgins JP Thompson SG Deeks JJ Measuring inconsistency in meta-analyses. BMJ (Clinical research ed) 2003;327:557–60. 10.1136/bmj.327.7414.557 PMC192859 12958120

[26] Shim S Yoon BH Shin IS Network meta-analysis: application and practice using STATA. Epidemiol Health 2017;39:e2017047. 29092392 10.4178/epih.e2017047 PMC5733388

[27] Mavridis D Giannatsi M Cipriani A A primer on network meta-analysis with emphasis on mental health. Evid Based Mental Health 2015;18:40–6. 10.1136/eb-2015-102088 PMC11234956 25908686

[28] Krahn U Binder H König J A graphical tool for locating inconsistency in network meta-analyses. BMC Med Res Methodol 2013;13:35–52. 23496991 10.1186/1471-2288-13-35 PMC3644268

[29] Tonin FS Rotta I Mendes AM Network meta-analysis: a technique to gather evidence from direct and indirect comparisons. Pharm Pract 2017;15:943. 10.18549/PharmPract.2017.01.943 PMC5386629 28503228

[30] Dias S Welton NJ Caldwell DM Checking consistency in mixed treatment comparison meta-analysis. Stat Med 2010;29:932–44. 20213715 10.1002/sim.3767

[31] Chaimani A Higgins JP Mavridis D Graphical tools for network meta-analysis in STATA. PloS One 2013;8:e76654. 24098547 10.1371/journal.pone.0076654 PMC3789683

[32] Salanti G Ades AE Ioannidis JP Graphical methods and numerical summaries for presenting results from multiple-treatment meta-analysis: an overview and tutorial. J Clin Epidemiol 2011;64:163–71. 20688472 10.1016/j.jclinepi.2010.03.016

[33] Peters JL Sutton AJ Jones DR Performance of the trim and fill method in the presence of publication bias and between-study heterogeneity. Stat Med 2007;26:4544–62. 17476644 10.1002/sim.2889

[34] Sweegers MG Altenburg TM Chinapaw MJ Which exercise prescriptions improve quality of life and physical function in patients with cancer during and following treatment? A systematic review and meta-analysis of randomised controlled trials. Br J Sports Med 2018;52:505–13. 28954800 10.1136/bjsports-2017-097891

[35] Cao PY Zhao QH Xiao MZ The effectiveness of exercise for fall prevention in nursing home residents: a systematic review meta-analysis. J Adv Nurs 2018;74:2511–22. 30043462 10.1111/jan.13814

