# Supplementary material for: The effectiveness of different exercise modalities on sleep quality: A protocol for systematic review and network meta-analysis
Source: Medicine (Baltimore). 2020 Jul 17;99(29):e21169. doi: 10.1097/MD.0000000000021169 (PMC7373514; doi:10.1097/MD.0000000000021169)
Supplement: Supplemental Digital Content [file medi-99-e21169-s001.docx]

| **PubMed** | | |
| --- | --- | --- |
| #1 | exercise[MeSH Terms] | 182153 |
| #2 | Search ((((((((exercises[Title/Abstract]) OR physical activit*[Title/Abstract]) OR training*[Title/Abstract]) OR danc*[Title/Abstract]) OR yoga[Title/Abstract]) OR taichi[Title/Abstract]) OR wuqinxi[Title/Abstract]) OR baduanjin[Title/Abstract]) OR yijinjing[Title/Abstract] | 508936 |
| #3 | #1OR#2 | 618310 |
| #4 | Search (((sleep[MeSH Terms]) OR sleep disorders[MeSH Terms]) OR sleep apnea syndromes[MeSH Terms]) OR ((sleep initiation and maintenance disorders[MeSH Terms])) | 134477 |
| #5 | Search insomnia [Title/Abstract] | 19487 |
| #6 | #4OR#5 | 143113 |
| #7 | #3AND#6 | 5518 |
| #8 | Search ((randomized controlled trial [pt] OR controlled clinical trial [pt] OR randomized [tiab] OR placebo [tiab] OR clinical trials as topic [mesh: noexp] OR randomly [tiab] OR trial [ti]) NOT (animals [mh] NOT humans [mh])) | 1144850 |
| #9 | #7AND#8 | 954 |
| **Web of Science** | | |
| #1 | TOPIC: (exercise) OR TOPIC: (physical activit*) OR TOPIC: (training*) OR TOPIC: (danc*) OR TOPIC: (yoga) OR TOPIC: (taichi) OR TOPIC: (wuqinxi) OR TOPIC: (baduanjin) OR TOPIC: (yijinjing) | 2795120 |
| #2 | (sleep) OR TOPIC: (sleep disorder) OR TOPIC: (sleep apnea syndrome) OR TOPIC: (sleep initiation and maintenance disorder) OR TOPIC: (insomnia) | 478876 |
| #3 | #1 AND #2 | 35228 |
| #4 | TOPIC: (randomized controlled trial) OR TOPIC: (controlled clinical trial) OR TOPIC: (clinical trial) OR TOPIC: (placebo) OR TOPIC: (randomized) OR TOPIC: (randomly) OR TOPIC: (blind*) | 3,018,284 |
| #5 | #3 AND #4 | 5198 |
| **EMBASE** | | |
| #1 | 'exercise'/exp OR 'physical activity'/exp OR 'training'/exp OR 'yoga'/exp OR 'dancing'/exp OR 'baduanjin'/exp | 728120 |
| #2 | exercises:ab,ti OR taichi:ab,ti OR wuqinxi:ab,ti OR yijinjing:ab,ti | 49255 |
| #3 | #1 OR #2 | 747605 |
| #4 | 'sleep'/exp OR 'sleep disorder'/exp OR 'sleep disordered breathing'/exp OR 'insomnia'/exp | 353552 |
| #5 | #3 AND #4 | 21226 |
| #6 | 'randomized controlled trial'/exp OR 'controlled clinical trial'/exp OR 'clinical trial'/exp OR 'placebo'/exp OR randomized:ab,ti OR randomly:ab,ti OR blind*:ab,ti | 2186360 |
| #7 | #5 AND #6 | 4343 |
| **CENTRAL** | | |
| #1 | MeSH descriptor: [Exercise] explode all trees | 22235 |
| #2 | exercises:ti,ab,kw OR physical activit*:ti,ab,kw OR training*:ti,ab,kw OR danc*:ti,ab,kw OR yoga:ti,ab,kw OR taichi:ti,ab,kw OR wuqinxi:ti,ab,kw OR baduanijn:ti,ab,kw OR yijinjing:ti,ab,kw (Word variations have been searched) | 154246 |
| #3 | #1 OR #2 | 155686 |
| #4 | MeSH descriptor: [Sleep] explode all trees | 5438 |
| #5 | MeSH descriptor: [Sleep Wake Disorders] explode all trees | 7153 |
| #6 | MeSH descriptor: [Sleep Apnea Syndromes] explode all trees | 2228 |
| #7 | MeSH descriptor: [Sleep Initation and Maintenance Disorders] explode all trees | 2165 |
| #8 | insomnia:ti,ab,kw(Word variations have been searched) | 9737 |
| #9 | #4 OR #5 OR #6 OR #7 OR #8 | 18165 |
| #10 | #3 AND #10 | 1642 |
| **PsycINFO** | | |
| S1 | MA exercise | 15290 |
| S2 | TI exercises OR AB exercises OR TI physical activit* OR AB physical activit* OR TI training* OR AB training* OR TI danc* OR AB danc* OR TI yoga OR AB yoga OR TI taichi OR AB taichi OR TI wuqinxi OR AB wuqinxi OR TI baduanjin OR AB baduanjin OR TI yijinjing OR AB yijinjing | 332688 |
| S3 | S1 OR S2 | 335030 |
| S4 | MA sleep OR MA sleep disorders OR MA sleep apnea syndrome OR MA ( sleep initiation and maintenance disorders ) | 29299 |
| S5 | TI insomnia OR AB insomnia | 11173 |
| S6 | S4 OR S5 | 36471 |
| S7 | S3 AND S6 | 1957 |
| S8 | MA randomized controlled trials OR MA controlled clinical trial OR MA clinical trial OR MA placebo OR TI randomized OR AB randomized OR TI randomly OR AB randomly OR TI blind* OR AB blind* | 179159 |
| S9 | S7 AND S8 | 284 |
| **SPORTDiscus** | | |
| S1 | SU exercise | 348218 |
| S2 | TI exercises OR AB exercises OR TI physical activit* OR AB physical activit* OR TI training* OR AB training* OR TI danc* OR AB danc* OR TI yoga OR AB yoga OR TI taichi OR AB taichi OR TI wuqinxi OR AB wuqinxi OR TI baduanjin OR AB baduanjin OR TI yijinjing OR AB yijinjing | 964547 |
| S3 | S1 OR S2 | 1056766 |
| S4 | MH sleep OR MH sleep disorders OR MH sleep apnea syndrome OR MH ( sleep initiation and maintenance disorders ) | 137000 |
| S5 | TI insomnia OR AB insomnia | 19615 |
| S6 | S4 OR S5 | 145393 |
| S7 | S3 AND S6 | 8500 |
| S8 | SU randomized controlled trials OR SU controlled clinical trial OR SU clinical trial OR SU placebo OR TI randomized OR AB randomized OR TI randomly OR AB randomly OR TI blind* OR AB blind* | 1201095 |
| S9 | S7 AND S8 | 1182 |
