# Supplementary material for: The effectiveness of different exercise modalities on sleep quality: A protocol for systematic review and network meta-analysis
Source: Medicine (Baltimore). 2020 Jul 17;99(29):e21169. doi: 10.1097/MD.0000000000021169 (PMC7373514; doi:10.1097/MD.0000000000021169)
Supplement: Supplemental Digital Content [file medi-99-e21169-s002.docx]

## Transformation formulas for estimating mean and standard deviation

(1) When calculating SD from M (confidence interval) for intervention or control group

a. the sample size in each group＞100

• SE= (upper limit-lower limit)/3.92

• SD=SE*N^ (1/2)

b. the sample size in each group≤100

• Input “tinv (1-0.95, N_1_-1)” in Microsoft Excel to obtain t

• SE= (upper limit-lower limit)/ t

• SD=SE*N^(1/2)

(2) When calculating SD from MD and P value between intervention or control group

a. reporting the exact p value

• Input “tinv (p, N_1_+N_2_-2)” in Microsoft Excel to obtain t

• SE=MD/t

• SD1=SE/N_1_^(1/2)

b. reporting only significant levels (e.g. P＜0.05 or P＞0.05)

• The conservative calculation is to take the upper bound P. (e.g. P＜0.05 is replaced 0.05)

(3) When calculating M (SD) from the m (interquartile range) for intervention or control group

a. the sample size＞25, M=m;

the sample size≤25, M=(𝑎+2m+𝑏)/4

b. the sample size≤15, SD=(((𝑎−2𝑚+𝑏)^2^/4+(𝑏−𝑎)^2^)/12) ^ (1/2)

the sample size 15<n≤70, SD=(𝑏−𝑎)/4

the sample size＞70, SD=(𝑏−𝑎)/6

(4) When calculating M (SD) from the m (interquartile range) for intervention or control group

a. M= (𝑞_1_ +𝑚+𝑞_3_)/3

b. calculation of SD

• Z=(0.25𝑁_1_+0.375)/(𝑁_1_+0.25)

• Input “norm. inv(1-z,0,1)” obtain μ

• SD=(𝑞_3_−𝑞_1_)/(2∗𝜇)

Note:

M= mean; SE=standard error of mean; SD=standard deviation; N=sample size; MD= mean difference; m=median; a= minimum value; b = maximum value; q1= first quartile; q3= third quartile
